# A feasible route for the design and manufacture of customised respiratory protection through digital facial capture

**DOI:** 10.1038/s41598-021-00341-3

**Published:** 2021-11-02

**Authors:** Luke N. Carter, Caroline A. Reed, Alexander P. Morrell, Anthony K. H. Fong, Rayyan Chowdhury, Ewan Miller, Federico Alberini, Balvinder Khambay, Shivana Anand, Liam M. Grover, Trevor Coward, Owen Addison, Sophie C. Cox

**Affiliations:** 1 School of Chemical Engineering, College of Engineering and Physical Sciences, University of Birmingham, Edgbaston, Birmingham, B15 2TT UK; 2 Centre of Oral, Clinical & Translational Sciences, Faculty of Dentistry, Oral & Craniofacial Sciences, King’s College London, Guy’s Hospital, London, SE1 9RT UK; 3 Department of Mechanical Engineering, College of Engineering and Physical Sciences, University of Birmingham, Edgbaston, Birmingham, B15 2TT UK; 4 School of Dentistry, College of Medical and Dental Sciences, University of Birmingham, Edgbaston, Birmingham, B15 2TT UK

**Keywords:** Health care, Occupational health, Biomedical engineering

## Abstract

The World Health Organisation has called for a 40% increase in personal protective equipment manufacturing worldwide, recognising that frontline workers need effective protection during the COVID-19 pandemic. Current devices suffer from high fit-failure rates leaving significant proportions of users exposed to risk of viral infection. Driven by non-contact, portable, and widely available 3D scanning technologies, a workflow is presented whereby a user’s face is rapidly categorised using relevant facial parameters. Device design is then directed down either a semi-customised or fully-customised route. Semi-customised designs use the extracted eye-to-chin distance to categorise users in to pre-determined size brackets established via a cohort of 200 participants encompassing 87.5% of the cohort. The user’s nasal profile is approximated to a Gaussian curve to further refine the selection in to one of three subsets. Flexible silicone provides the facial interface accommodating minor mismatches between true nasal profile and the approximation, maintaining a good seal in this challenging region. Critically, users with outlying facial parameters are flagged for the fully-customised route whereby the silicone interface is mapped to 3D scan data. These two approaches allow for large scale manufacture of a limited number of design variations, currently nine through the semi-customised approach, whilst ensuring effective device fit. Furthermore, labour-intensive fully-customised designs are targeted as those users who will most greatly benefit. By encompassing both approaches, the presented workflow balances manufacturing scale-up feasibility with the diverse range of users to provide well-fitting devices as widely as possible. Novel flow visualisation on a model face is presented alongside qualitative fit-testing of prototype devices to support the workflow methodology.

## Introduction

The COVID-19 pandemic has highlighted the need for effective respiratory personal protective equipment (PPE), particular FFP3/N99 filtering standards, that may be comfortably worn by front-line workers for prolonged periods. At the time of writing, alert levels in most countries show the virus in ‘general circulation’, and with vaccine deployment in the early stages alongside the threat of further mutations, front-line workers face an ongoing need for filtering PPE. On 3rd March 2020, a statement by the World Health Organisation^1^ called for a 40% increase in PPE manufacturing worldwide to meet growing demand, warning of serious threat to health and life of frontline workers without adequate supplies of effective face-masks and respirators. Acquisition of respiratory PPE has become globally competitive with many local jurisdictions concerned about supply availability^2^. Against this backdrop this research aims to address the persistent problems of poor respirator fit rates and user comfort by proposing a system exploiting existing accessible digital technologies to rapidly capture and process facial topographies. We further demonstrate the ability to automate parameterisation enabling individual fit selection and/or customisation of a new respirator device.

Assuming appropriate filtering material, typically melt-blown polypropylene, effectiveness of a respirator device is largely dictated by the fit or seal it forms with the user’s face. Quantitative and qualitative physical test methods are both used during respirator selection; the former provides superior sensitivity and the latter a robust simplicity^3^. Both are time consuming for larger workforces^4^. Additionally, manufacturers recommend users performs a ‘fit check’ upon every donning, by covering the filter and inhaling to feel for a good facial seal. The reliability of this method is questionable with Lam et al*.*^5^ reporting accuracy of self-checks between 57.5 and 70.5% compared against quantitative results. Likewise, fit test failure rates have been widely studied with pass rates varying wildly depending on devices tested, testing methods, and usage situation. Shaffer and Janssen^6^ reported pass rates of 0–88% across ten studies examining different N95 models in a review highlighting the extent of the problem. An investigation by Zhuang et al*.*^7^ examined 101 different respirators using 25 subjects. Only 32% of the devices achieved acceptable fit in at least one of three donnings for > 75% of participants. Burgess amd Mashingaidze^8^ reported a failure rate of 69% in devices across ten pharmaceutical companies with success shown not to be associated with usage frequency, years of experience, or user training. Further data by Foereland et al*.*^9^ showed a more promising 62% pass rate for 701 fit tests across 14 different respirators in the smelting industry. It also highlighted pass rates of 92% and 100% for the only silicone-to-skin interfacing respirators with changeable filters included in the study.

User gender is a notable factor in the fit success rates drawing into question the gender and racially blended anthropometric datasets currently used for respirator design and standard testing. McMahon et al*.*^10^ reported 95.1% fit success for male participants compared with 85.4% for females and highlights that younger women are more likely to fail fit tests. One design in the study by Lam et al*.*^5^ revealed pass rates of 72.2% vs. 58.1% for males and females respectively and 63% for males compared with 56% for females in the study by Foereland et al*.*^9^.

These overall fit test failure rates highlight the extent of the problem, but do little to clarify where and why respirator fit has failed. Only a limited number of studies examine the location of leaks in the facial seal. In a study by Brosseau^11^, the most common sites were the nosepiece (30%), chin (6%), and cheeks (4%). Similarly, Oestenstad and Bartolucci^12^ showed the most common leak sites reported were nose and cheek (24.9%), cheek (19.8%), and nose (14.3%). This supports anecdotal evidence from users and clinicians that the nose presents a specific problem region due to the wide variety in user’s nasal profile and lack of soft tissue able to accommodate mismatch between a standard respirator nose form and the user.

One further consideration of widespread respiratory PPE use is user comfort. Throughout the COVID-19 pandemic, images on social media have highlighted the bruising and abrasions that can result from prolonged wearing of disposable PPE, particularly designs with deformable metal nose clips, an issue that is supported by user focussed studies. Locatelli et al*.*^13^ present reports from health workers during a focus group setting with direct statements that respirators leave deep indentations on the user’s face, cause skin irritation, that the metal nose bars pinch, and that fitted masks are too tight. Even more concerning is acknowledgement by users that wearing respiratory protection interferes with patient care both directly from distraction due to the discomfort, and also due to ‘rushing’ when the user feels as though the facial seal is poor. A study by MacIntyre et al*.*^14^ reported 52.2% of users (366/701) complained of pressure on the nose during respirator usage and 59% of participants in a study presented by Radonovich et al*.*^15^ discontinued respirator use within an 8 h period due to discomfort.

Given this background, it is clear a fitted or customised approach to respirator design may dramatically improve the fit, comfort, and ultimately the level of protection that a user receives from their respiratory PPE. 3D printing is a well-positioned technology to support this demand having already been highly visible in the global COVID-19 response^16^. The concept of customisation using 3D digital data is not new. In 1997 a U.S. military report^17^ discussed applying laser 3D scanning to customise manufacture, fit, and model helmets and face-pieces worn by military personnel. More recently a rigid polymer proof-of-concept respirator device was customised using 3D scan data by Swennen et al*.*^18^. It demonstrated a viable method of decentralised respirator production given local shortages of mass-produced PPE, however acknowledged that the rigid design may cause skin abrasions during extended use and does not present data to verify the technique’s efficacy. Likewise, Cai et al*.*^19^ proposed a user specific rigid polymer interface device for use with traditional N95 masks in an effort to improve comfort. They show a reduced point-load on the user’s face when using the device under a neutral expression, but more severe loads with different user expressions potentially due to the rigid nature of the material. These issues were addressed it a pilot study by Makowski and Okrasa^20^. Their fully-customised 3D printed respirator was produced using an elastomeric polymer allowing for some level of compliance with the subject’s face. The prototype not only met the relevant standards for such a device but showed significantly improved user comfort and did not cause bruising or skin irritation. It highlights the importance of considering the individual user’s face when evaluating the effectiveness of respiratory protection and, in the authors’ words, shows “A good quality respirator equipped with high-efficiency filters is not nearly enough to ensure a proper protection of workers”. A further novel and automated approach to move directly from stereophotogrammetry scan data to a customised design was demonstrated by Shaheen et al.^21^ combining the use of traditional facial landmarks and direct mapping of the facial contour to form the device design. The only notable drawback of these two approaches is the need to customise and produce the entire device for each user which adds significant time and cost to the process thus limiting the potential for scale-up.

The current research presents a method to rapidly capture and quantify key features from a user’s facial profile using non-invasive optical imaging. Key facial parameters drive the design and selection of a semi-customised or fully-customised prototype respirator design as appropriate. Combining these innovative customisation techniques and intelligent material choice, the fit and comfort of these devices is improved for individual users whilst standardising production where possible to give a feasible larger scale solution.

## Methods

### Process overview

**Figure 1:**
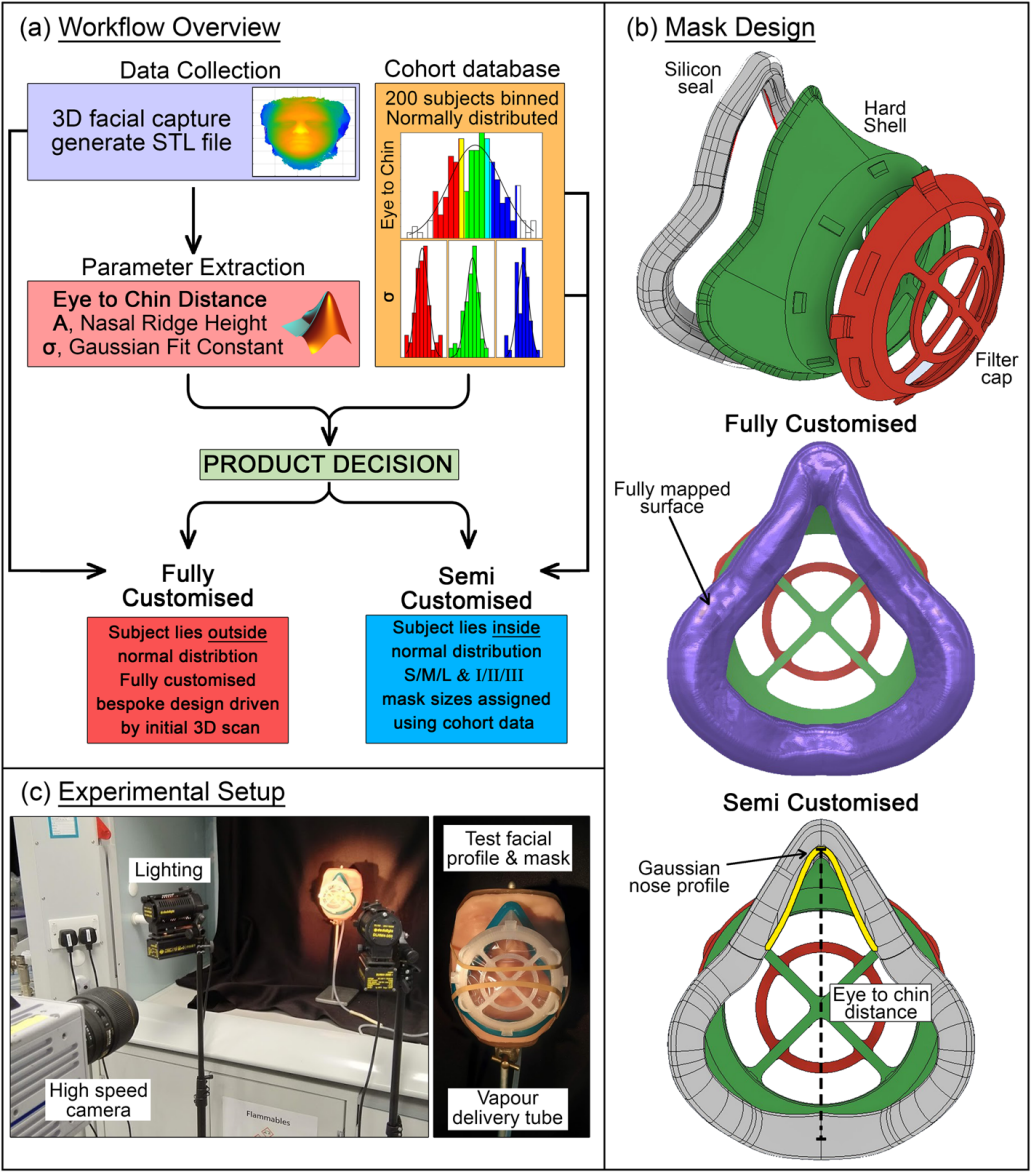
(**a**) Workflow from scan to product, (**b**) mask design highlighting fully customised and semi-customised aspects, (**c**) experimental setup for flow visualisation used to rapidly assess fit.

Through examination of commercial designs and discussions with end-users regarding problems associated with disposable respirator models, a workflow has been developed to provide improved fit and comfort in a new reusable ‘3-part’ mask design; an overview of this workflow is shown in Fig. 1a. 3D facial scan data is collected using one of several possible methods. Bespoke post-processing routines extract dimensional parameters from the face including the distance from eyeline to the chin (eye-to-chin distance) and parameters defining a Gaussian fit of the subject’s nasal profile. These facial parameters are used to categorise the user in to one in a set of mask designs initially defined by examining distributions in a sample cohort. The analysis will inform the user which of these semi-customised designs is suitable to achieve a good fit. Should the user’s facial parameters lie outside those of the semi-customised set, then a fully customised approach driven by the initial scan data will be recommended.

The general design is shown in Fig. 1b consisting of a rigid polymer hard-shell defining the overall shape, flexible silicone seal providing compliant interface to the user’s face, and front cap to securely hold standard filter material. It is intended to be simple to manufacture and disinfect between uses.

Overall size of hard-shell and seal is scaled according to the user’s eye-to-chin distance in the semi-customised approach and the nasal profile of the silicone seal follows a Gaussian curve as indicated in Fig. 1b. The fully customised approach features a silicone seal with surface fully mapped to the user’s facial 3D scan data.

The novel bespoke test rig shown in Fig. 1c was constructed to assess the efficacy of the two design methodologies against a model facial profile via flow visualisation and high-speed photography.

### Facial data

#### Sample selection

A cohort of 100 volunteer participants working in a clinical academic centre during April–May 2020 of mixed facial form, gender and ethnicity were included (King’s College London Institutional Ethics Approval MRPP-19/20-18570). This dataset was combined with a further random sample of 100 three-dimensional facial images of staff and students at the University of Leeds and originated from a larger data set previously analysed for dynamic facial asymmetry^22^ (Dental Research Ethics Committee (DREC) approval (240915/BK/179)). All participants had no previous history of facial surgery or trauma, were aged between 18 and 40 years of age and had symmetrical faces and normal occlusions. Facial images were analysed and only numerical data was generated and shared, in the form of an anonymised spreadsheet. As part of the two listed ethical approval processes, informed consent was obtained from all participants and all methods were performed in accordance with the relevant guidelines and regulations.

For this feasibility study no formal sample size calculation was performed for the combined 200 participants. Accordingly quantitative measurements should not be generalised beyond this specific study although the process feasibility and approach is likely to be valid given the strong accordance with key average facial topographical measurements in the collected sample population with the United States Centre for Disease Control anthropometric averages, against which the developed algorithms for sizing selection were tested.

#### Facial capture

Images of volunteers were captured using a stereophotogrammetry three-dimensional (3D) image capture and analysis system (Dimensional Imaging (DI4D), Glasgow, Scotland). Four linked cameras capture simultaneous photographs of the patient which is then reconstructed in to a 3D surface and exported in stereolithographic (*.stl) format. A subset of 9 volunteers from King’s College London were also imaged in the same session using the Bellus3D application using Apple iPhone X TrueDepth camera in order to compare DI4D and Bellus3D scan methods.

#### Facial parameter extraction

Extracting key parameters from facial scan data allows for rapid evaluation of each large and cumbersome scan dataset. Eye-to-chin distance was selected to evaluate facial height being representative of the typical span in a well-fitting device. Likewise, a Gaussian curve fitted to the 2-dimensional nasal profile extracted perpendicular to the nasal ridge describes the general height and width of a well-fitting respirator nose form in two simple numeric parameters, ‘A’ and ‘σ’ respectively where:

1 $$Standard\;form\;of\;a\;Gaussian\;curve{:}\quad f\left( x \right) = Ae^{{ - \frac{{x^{2} }}{{2\sigma^{2} }}}} .$$

**Figure 2:**
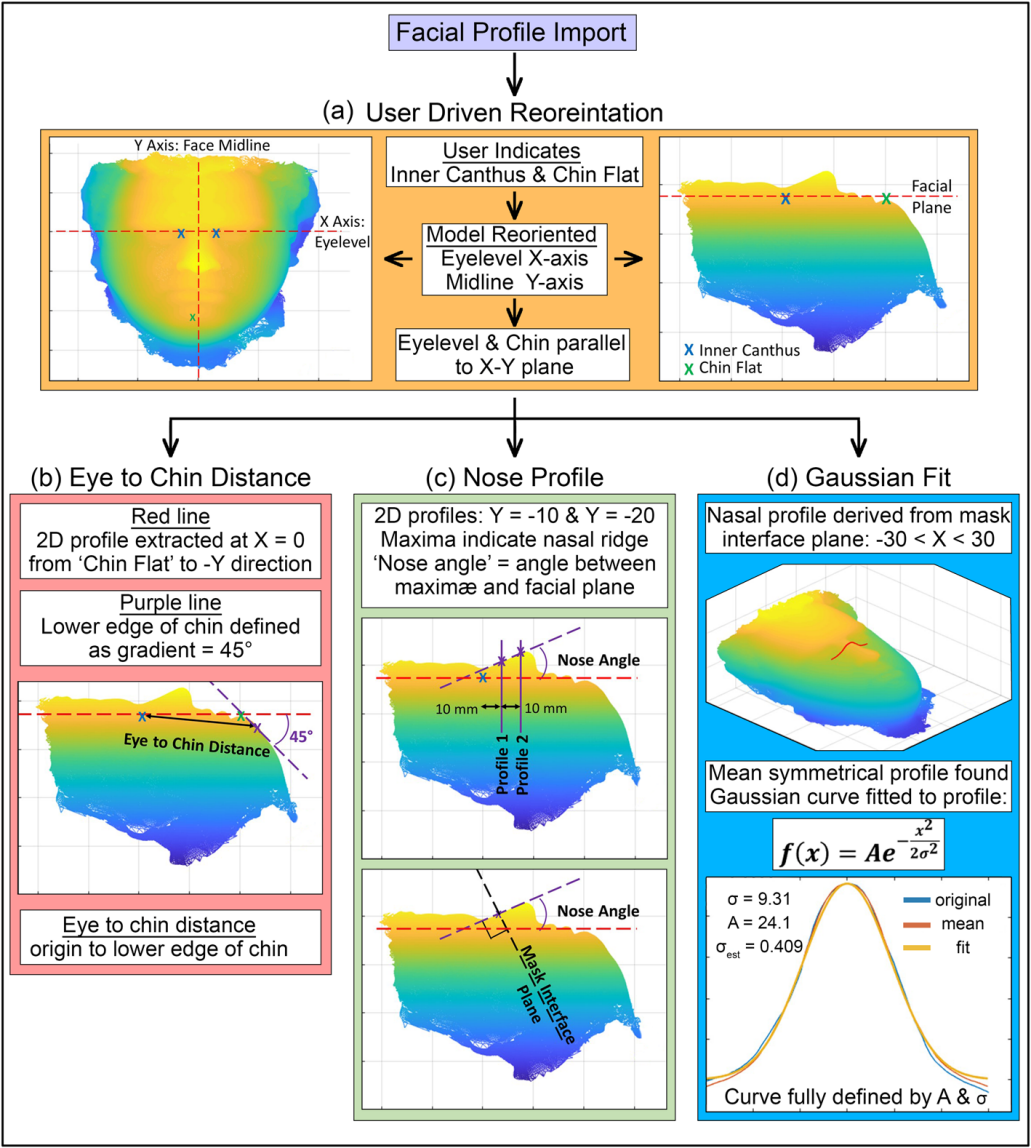
Diagram showing facial scan data processing logic for key parameter extraction as applied within the custom MATLAB script. (**a**) Shows the orientation stage with blue crosses indicating user input and red dashed lines showing the relevant axes/planes. (**b**) Illustrates the method for eye-to-chin distance measurement. (**c**) Illustrates nose profile extraction (**d**) shows the Gaussian fit to the extracted nose profile.

This evaluation was performed by a bespoke MATLAB^23^ script. Initial user registration informs the correct orientation of the scan data before the three key parameters are extracted. The full logic of this operation is presented schematically in Fig. 2 and MATLAB source code is available on request to the authors.

#### Data analysis

Extracted facial parameters from the volunteer cohort using scanning methods from both institutions were used to determine the semi-customised mask sizes. Categories were established in a two stage process; firstly a normal distribution was fitted to the eye-to-chin distance of the full cohort. Three hard-shell sizes corresponding to eye-to-chin distance were centred on the lower full width half maximum (FWHM) boundary, maximum, and upper FWHM boundary (‘Small’, ‘Medium’, ‘Large’). A similar process was performed for distributions of σ values in each size subset to determine the three nose profiles (I, II, III) for each hard-shell size. Thus nine different semi-customised mask designs were established. Corresponding mean ‘*A*’ values were calculated for the subsets to fully define each nasal profile.

### CAD development

Computer aided design (CAD) models and methods were developed for both the semi-customised and fully customised design approaches.

#### CAD customisation: semi-customised

**Figure 3:**
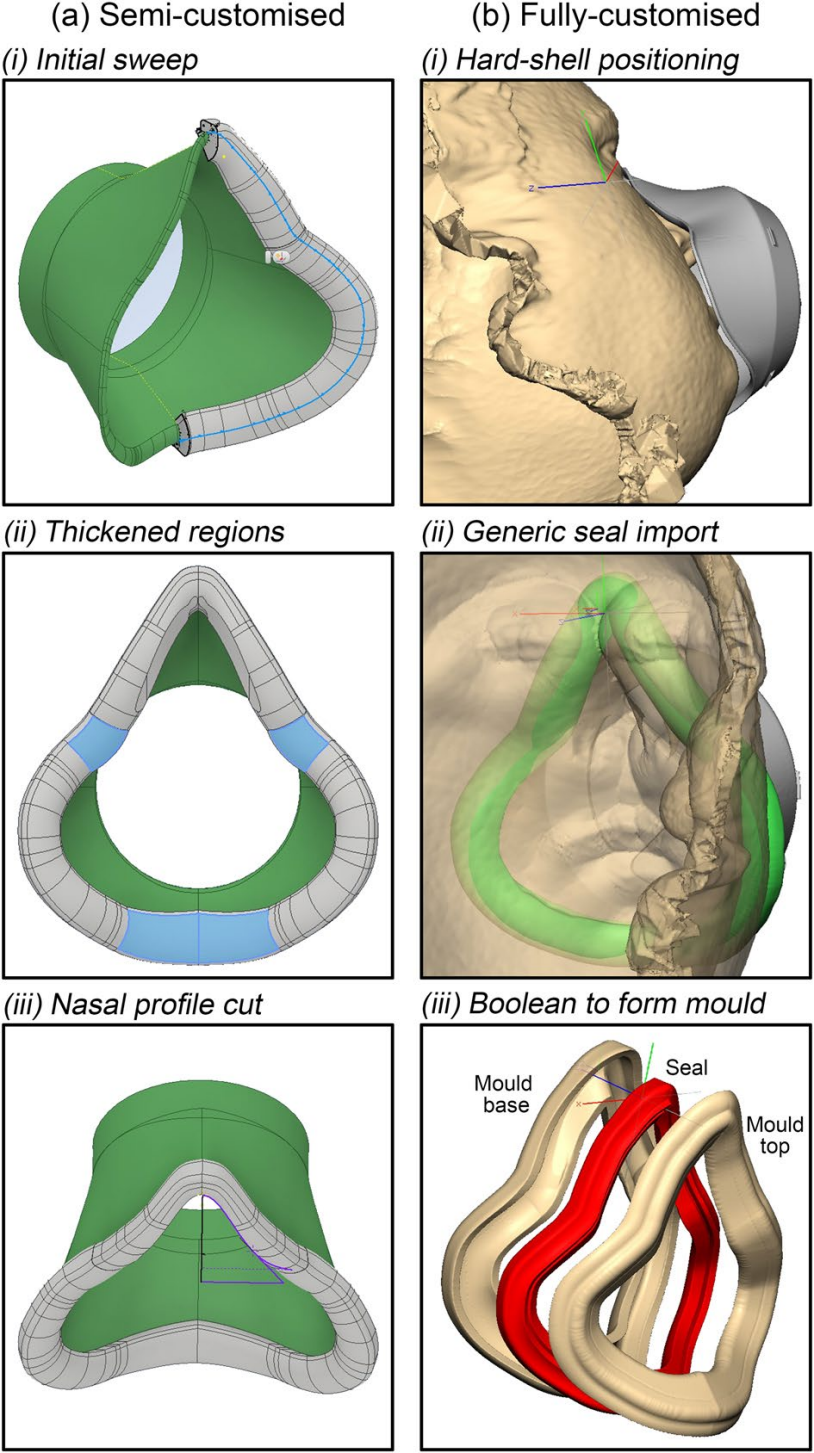
CAD Methodology (**a**) Shows the semi-customised seal design method from the (i) initial sweep, (ii) thickened regions, and finally (iii) cut to form the nasal profile. (**b**) Shows the fully-customised route; (i) initial alignment and offset of the hard-shell from the facial scan data (ii) import of a generic seal, and (iii) formation of the mould.

CAD for the semi-customised design was developed using a combination of Autodesk Inventor^24^ and Autodesk Fusion 360^25^ as appropriate. The facial interface profile of the hard-shell was designed to be scaled according to eye-to-chin distance whilst keeping the filter geometry constant enabling common filter cap geometry and filter area across all design variants. Seal geometry was created using a combination of sweep and loft operations following the hard-shell profile (Fig. 3a(i)) with a final Boolean subtraction of the hard-shell to ensure a good mate between the two components. In general, a 5 mm of silicone thickness was specified between the face and hard-shell with initial prototyping advising slight thickening of the seal (1–3 mm) in the region of the user’s maxilla and chin (Fig. 3a(ii)). Minimum silicone thicknesses (2 mm) were exceeded in all regions to ensure manufacturability. Finally, the Gaussian nasal profile defined by the *A* and *σ* parameters was cut from the inner contour of the seal at the mean nasal ridge angle from the initial cohort, 25°, to the facial plane (Fig. 3a(iii)).

#### CAD customisation: fully customised

A fully customised design approach was established to accommodate the small minority of users falling outside the standard semi-customised sizes and two fully-customised seals were produced for testing via this method.

Subject facial scan data was imported in to Geomagics FreeForm Plus 3D^26^ software. Appropriately sized hard-shell were also imported and translated onto the virtual face with a minimum 5 mm offset between the two to accommodate the seal (Fig. 3b(i)). A generic seal model was imported around the perimeter of the hard-shell and an operator ensured that it intersected the facial profile around the entire perimeter (Fig. 3b(ii)). Finally a Boolean subtraction was performed to create the customised surface of the seal and mate with the generic hard-shell design.

#### Seal mould CAD

Both customisation approaches used the same methodology to produce seal mould geometry. A CAD body was created by offsetting the outer surface by 4 mm and subtracting the seal geometry by Boolean operation. Finally, the mould was sectioned around its perimeter to create the two halves ensuring that no undercuts exist which would inhibit part removal (Fig. 3b(iii)).

### Manufacture of prototypes

#### Rigid components

Hard-shell and respirator cap components were 3D printed using a Connex 1 Objet 260 Polyjet Printer (Stratasys, UK) in UV cured resin, RGD720 (UTS 50–65 MPa, Elastic Modulus 2000–3000 MPa^27^), with 16 µm layer thickness and 600 dpi x/y resolution. Standard dissolvable support structures were removed following production using water-jet during post-processing.

#### Silicone seals

Seal moulds were 3D-printed from the thermoplastic polymer, polylactic acid (PLA), by fused filament fabrication (Cubicon Single Plus 3D, iMakr, UK). The system was fitted with a 0.4 mm diameter nozzle operating at 210 °C and a layer thickness of 0.1 mm. They were produced and used without further post-processing. Completed moulds were sprayed with a wax based release spray (MACWAX, Technovent, UK) to facilitate silicone removal.

All seals were made using a biocompatible silicone, M511 (Technovent, UK), traditionally used in the manufacture of facial/body prostheses and was shown to reliably cure in a representative mould during initial tests. It was softened from 25 to 15 shore with the addition of M513 (10% vol.) softening agent providing a balance between compliance and structural stability. Moulds were manually packed, secured with cable ties and processed in a pressure chamber; 3 h, 55 °C, 2 bar. Following demoulding, silicone trim was manually removed with a blade.

### Characterisation

To evaluate the fit of each mask design, a novel flow visualisation methodology was developed.

The respirator filter opening is sealed using a polythene sheet and fitted to a demonstration facial profile. A glycerol vapour (90% glycerol, 10% water) is injected in to the mask via an aperture approximately located at the profile’s mouth. The vapour fills the mask volume and may be observed escaping in areas of poor or discontinuous seal. High speed photography records the process and post-processing is used to qualitatively and semi-quantitatively evaluate the seal.

A single demonstration facial profile was produced and used for all tests. It consisted of 4 mm thick silicone layer, analogous to facial tissue, supported by a rigid polymer 3D printed base to better represent the overall compliance of a real face. Silicone was moulded and formed using the same general method and specification to that used for the seal production.

Flexible tubing was connected to the rear of the facial profile to enable injection of glycerol vapour. The facial profile was positioned on a stand and illuminated using two spot-lights in front of a matt black background within a fume hood as shown in Fig. 1c. High-speed footage was recorded using a high-speed camera (Photron Fastcam SA3, CA, USA) at 250 f.p.s. and with a shutter speed of 500 µs. Maximum illumination was used to allow the lens aperture to be adjusted to f/11 and provide the greatest possible depth of field. Recording was initiated approximately 1 s before manual vapour injection and allowed to continue for approximately 10 s for each test.

Post-processing was via bespoke MATLAB script. Briefly, greyscale values from the first five frames were averaged to create a background image intended to mitigate minor variations between frames. Mean images across ten frames were derived from the raw footage every 25 frames (0.1 s) and subtracted from the background image. A threshold was applied with pixels with an absolute deviation of < 3 assumed to be showing no deviation from the background and a simple despeckle filter used to reduce noise. Finally, values were scaled by a simple factor between 1 and 2 according to corresponding background value in an attempt to accommodate for inconsistent brightness (i.e. Difference between facial profile and matt black background). Resulting deviation values were visualised using a false colour palette and super-imposed on to the background image to produce an animation showing qualitative vapour intensity throughout the experiment. For each processed frame a simple pixel count was performed to provide a semi-quantitative measure of vapour escape against time.

Three of the semi-customised designs, ‘Small’(II)/’Medium’(II)/’Large’(II), were tested along with the no seal condition, a fully customised seal, and an incorrectly customised seal (i.e. customised to facial scan data other than that of the test profile).

### Prototype fit testing

A subset of clinician volunteers who had a record of failures in fit-testing of multiple commercially available soft-interface N99 half-mask respirators (both PureFlo 1000 (Gentex Corporation, USA) and 3 M 6200, (3 M, USA)) were scanned using DI4D and assigned prototype respirators according to our developed pipelines. Devices were qualitatively fit-tested and evaluated for a peripheral seal^3^.

### Use of facial images

**Figure 4:**
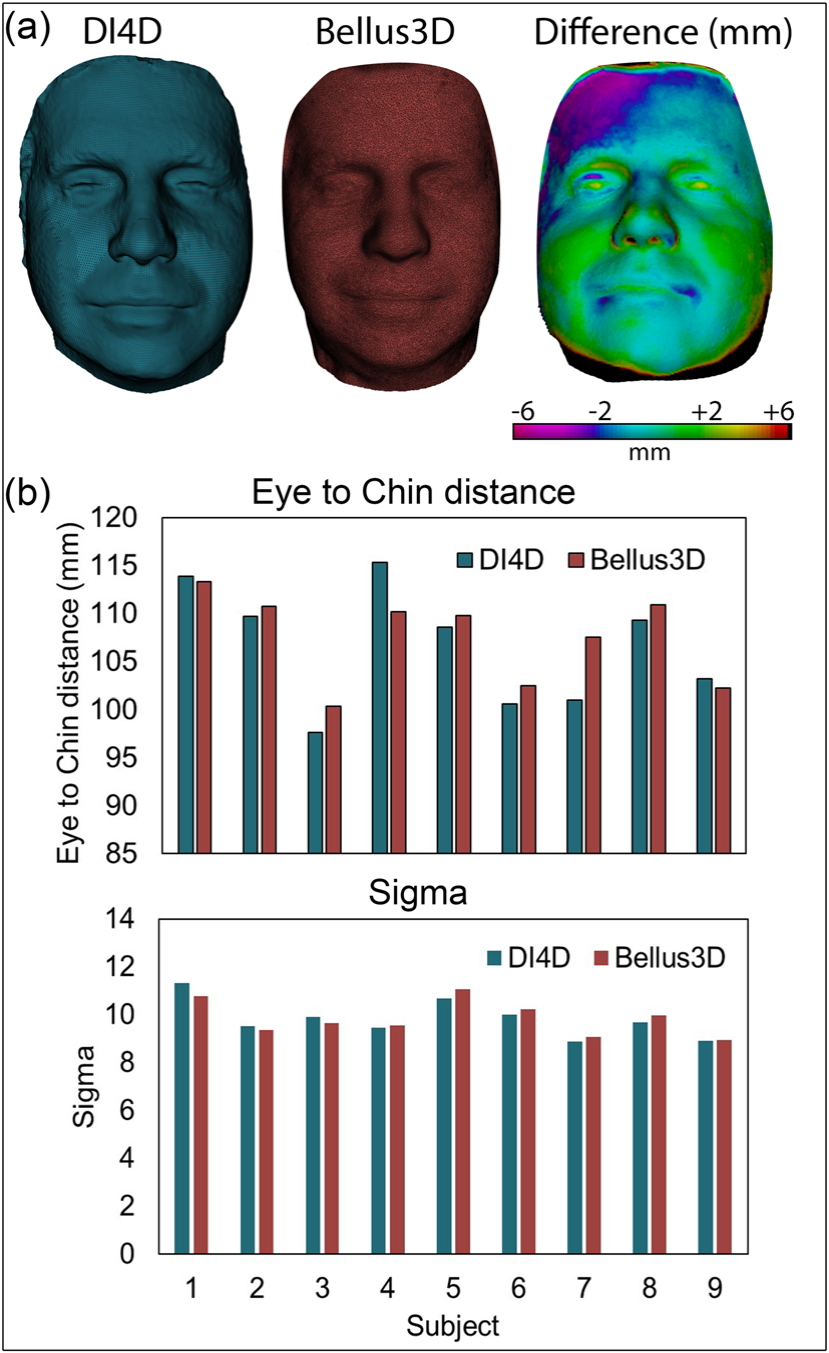
Comparison of DI4D and Bellus3D scanning method showing (**a**) typical false colour direct comparison highlighting deviation between the two techniques for a subject and (**b**) the extracted measurements of eye-to-chin distance and ‘σ’ from two different scanning methods of nine subjects.

Informed consent was gained for publication of the images shown as illustrative scan data in Figs. 1, 2, 3, and 4 and those used as exemplars of mask fit in Fig. 7.

## Results and discussion

### Scan method evaluation

Comparison of the nine subjects scanned using both DI4D and Bellus3D methods is shown in Fig. 4. Meshes for a typical subject generated using both methods are pictured in Fig. 4a alongside a false colour image showing straight line deviation between the DI4D and Bellus3D data. Accuracy of the Bellus3D method tends to decrease radially around the face compared to the ‘Gold Standard’ of the DI4D system, however the central features used to extract the key parameters of eye-to-chin distance and nasal profile are seen to only deviate by approximately ± 2 mm between scanning methods.

Figure 4b shows the extracted facial parameters, eye-to-chin distances and ‘σ’, for the nine subjects using both scan methods. It shows no notable trend in deviation between the two methods and indicates only small variations in the extracted parameters. For eye-to-chin distance, the mean difference is 2.4 mm (2.25%) with a standard deviation of 2.9 mm. Likewise for ‘σ’, the mean difference is 0.22 (2.18%) with a standard deviation of 0.26. This generally good agreement resulted in the same semi-customised design assigned to each of the nine subjects regardless of scan method.

This comparison supports the concept that user scanning and fitting using the semi-customised approach could effectively be conducted in a decentralised way without the need for specialised equipment. Whilst the iPhone 11 does represent a higher end consumer device, it is likely that one could be available within a wide range of settings where respirator fitting is required. Scanning using a familiar device would allow for it to effectively be carried out by non-specialists locally without the need for further training, travel, or expense.

### Sample set data analysis

**Figure 5:**
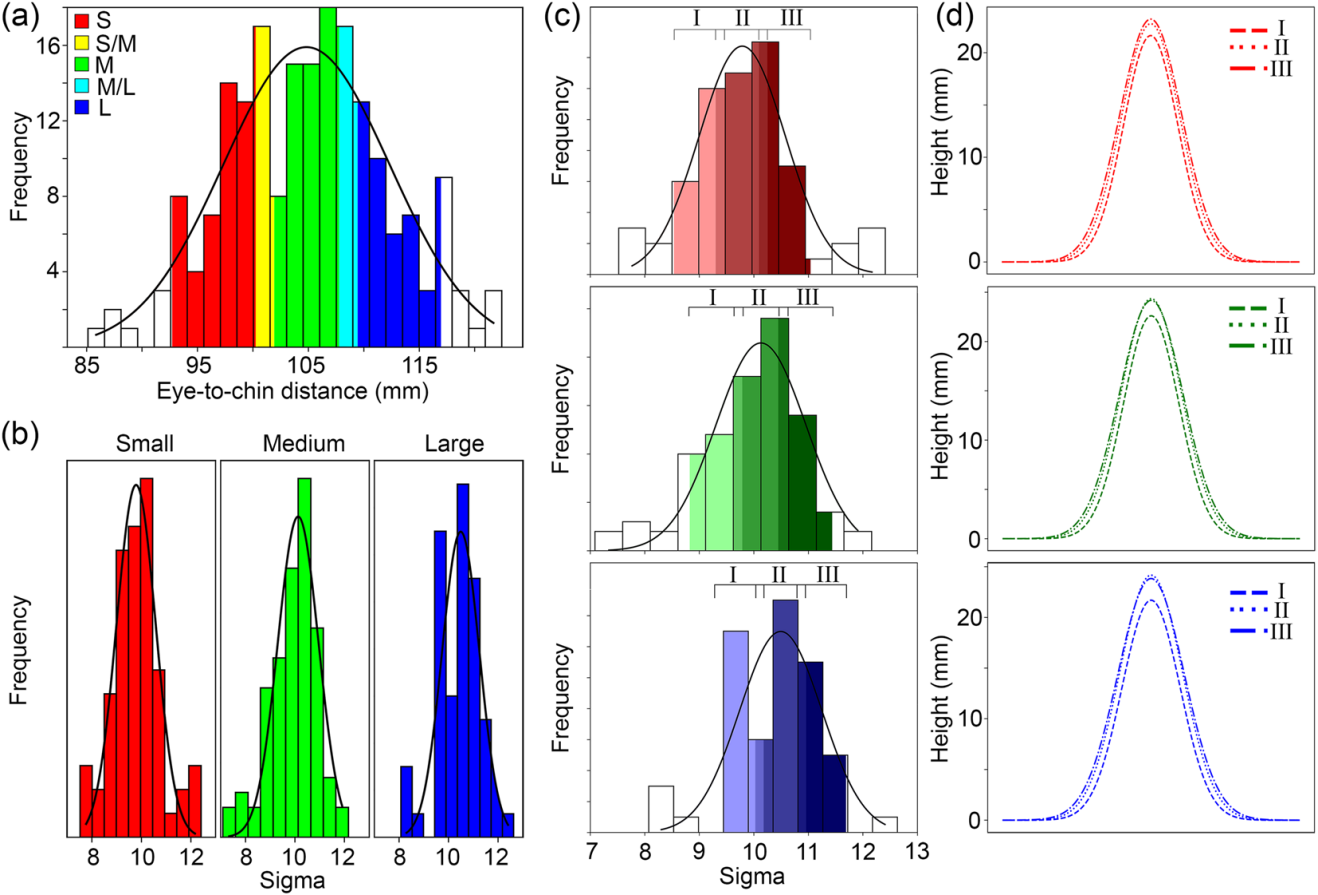
Graphs showing the extracted facial parameters from the sample cohort of 200 and subsequent determination of semi-customised designs. (**a**) Shows distribution of the eye-to-chin distance of the full 200 subjects with colour representing the different corresponding hard-shell sizes (S/M/L). (**b**) Shows the distribution of σ values for each hard-shell size and (**c**) shows the corresponding nasal profiles (I, II, III) for each. (**d**) Illustrates the nasal profiles established for each of the semi-customised designs.

Figure 5 shows the extracted facial parameters for the sample cohort and corresponding determination of the nine semi-customised designs as described in “Facial data”. The full distribution of eye-to-chin distances is shown in Fig. 5a with the three hard-shell size categories, ‘Small’/’Medium’/’Large’, centred around the lower FWHM boundary, maximum, and upper FWHM boundary of the fitted normal distribution respectively. Small overlaps at the Small/Medium boundary and Medium/Large boundary are shown in yellow and cyan respectively. These regions allow for some flexibility during mask assignment with borderline users potentially belonging in either category. The cumulative range across all hard-shell sizes includes 87.5% of subjects with the remaining 12.5% located at either extreme. Users in these outlying regions would be flagged as candidates for the fully-customised route.

Similar normal distributions were fitted to ‘*σ*’ values within the hard-shell size categories as show in Fig. 5b. These distributions were categorised in a similar way to that of the eye-to-chin distance to give three nasal profile ranges, I/II/III, for each hard-shell shown in Fig. 5c. Finally, corresponding mean ‘*A*’ and *σ* values for each *σ* range were calculated resulting in nine unique nasal profiles for the semi-customised designs shown in Fig. 5d. Range limits and parameters defining each of the nine designs are provided in Table 1.

**Table 1:** Ranges and mean values of eye-to-chin distance, ‘σ’, and A for each of the nine semi-customised designs.

Hard shell	Eye-to-chin (mm)			Nasal profile	Sigma, σ			A (mm)
	From	To	Midpoint		From	To	Mean	Mean
Small	92.8	101.8	97.3	I	8.5	9.5	9.1	21.6
				II	9.3	10.3	9.9	22.8
				III	10.1	11.1	10.4	23.2
Medium	100.3	109.4	104.9	I	8.8	9.8	9.3	22.6
				II	9.6	10.6	10.2	24.4
				III	10.5	11.5	10.7	24.2
Large	107.8	116.9	112.4	I	9.3	10.2	9.7	21.7
				II	10.0	11.0	10.5	24.2
				III	10.8	11.7	11.2	23.8

These distributions form the basis of the semi-customised sizing process. Parameters from a user are first assigned an overall hard-shell size based on eye-to-chin distance and then a corresponding fitted nasal profile based on *σ* value. Users falling in to one of the overlap regions are assigned hard-shell size based on the closest *σ* match in either category. For example, an eye-to-chin distance of 100.5 and σ of 11.2 would yield a Small/Medium mask size which is closer to Small. However, as *σ* is too large to fit within the Small group a Medium (III) mask is selected instead. Users falling outside the category limits at either stage are flagged for the fully-customised route.

3D scanning provides a rapid and increasingly available method of capturing the shape of a user’s face at a useful resolution made possible by growing sophistication in consumer devices. Unfortunately the resulting datasets are often large and unwieldy making their use in customisation labour intensive. By extracting facial parameters using the methods outlined here, each 3D facial scan can be rapidly evaluated using a few simple values. In this case aspects of respirator design, namely the overall size and nasal profile, that have been reported to critically impact device effectiveness^11,12^ are efficiently described in three numbers. Data analysis of the cohort demonstrates that a large proportion of users can be categorised in to a relatively small number of designs showing potential to improve fit and function whilst maintaining viability of large scale manufacture. Identifying outliers in this process recognises that the variety and diversity in user’s faces will inevitably make any level of standardisation impossible for some and directs potential manufacturers to target labour intensive, fully-customised, devices at those who truly need them.

### Flow visualisation

**Figure 6:**
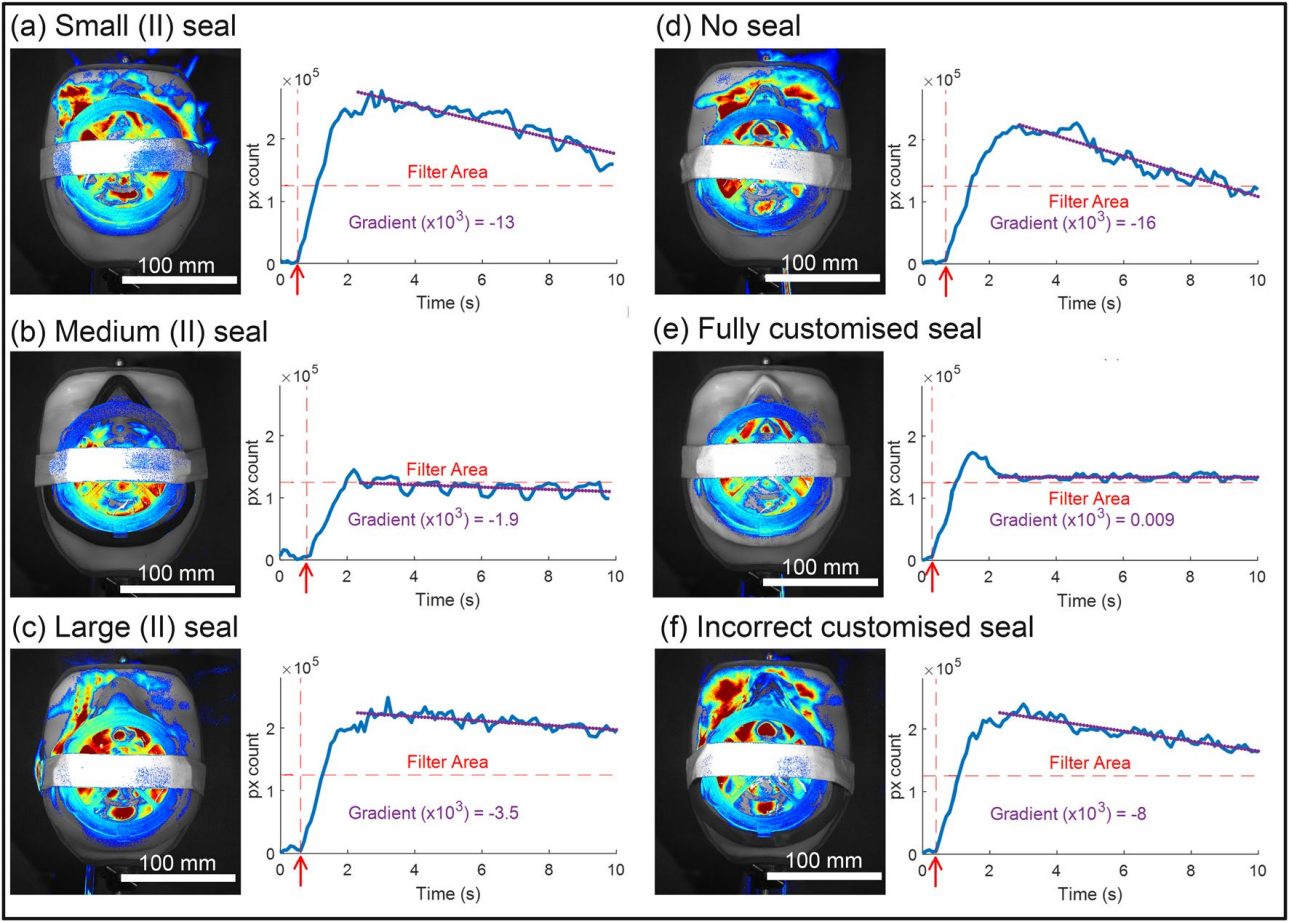
Figure showing typical false colour vapour visualisations for (**a**) ‘Small’(II), (**b**) ‘Medium’(II), (**c**) ‘Large’(II), (**d**) no seal condition, (**e**) fully customised seal, and (**f**) an incorrectly customised seal. Alongside each is resented the corresponding vapour pixel count against time for the fully analysed video. Horizontal dashed lines indicate a pixel count corresponding to the filter area, vertical dashed lines (red arrow) indicate the moment of vapour injection.

Figure 6 shows typical false colour vapour visualisation frames for each of the mask designs tested. All visualisations show vapour within the mask observed through the sealed filter opening and translucent body. Facial parameter extraction categorised the test facial profile was categorised as ‘Medium’(II). Both the ‘Medium’(II) (Fig. 6b) and the fully customised designs (Fig. 6e) showed good seal performance with minimal vapour release. All other designs showed extensive and rapid vapour loss; ‘Small’(II) (Fig. 6a) design appeared to have severe leaks around the nose whereas the ‘Large’(II) (Fig. 6c) design appeared to show vapour escape along the sides adjacent to the cheeks. This dramatically highlights the need to for a good fit both in size and nasal profile to ensure device effectiveness.

Adjacent plots in Fig. 6 show the vapour pixel count against time indicating severity of vapour loss. Vertical dashed lines (arrows) indicate the moment of the vapour injection and horizontal dashed lines show the approximate count relating to vapour observed through the translucent filter area. Both the ‘Medium’(II) (Fig. 6b) and Fully Customised (Fig. 6e) designs stabilise at a count similar to the filter area following an initial transient phase suggesting that the mask volume fills with vapour but allows very little to escape thus indicating a good seal.

Following the initial transient phase a linear fit (least-squares) was applied to the count data to semi-quantitatively assess the severity of the leak. Large gradients indicate all vapour leaving the mask volume very quickly. Smaller gradients suggest a longer sustained release and comparatively better seal. By this method designs rank best to worst in the order: fully-Customised, ‘Medium’(II) (*selected design*), ‘Large’(II), Incorrectly-Customised-Seal, ‘Small’(II), and No Seal.

These results not only validate the semi-customised approach of approximating the user’s nasal profile to a Gaussian curve, but also the selection of silicone as appropriate seal material. Its compliant properties have previously been reported to improve respirator fit rates^9^ and here it is shown to be effective at accommodating the small mismatch between the user’s nasal profile and Gaussian fit.

Whilst providing a useful rapid evaluation method, this experimental setup has some limitations. Firstly, vapour injection is manual and, whilst the operator aims for consistency, the amount of vapour used in each experiment may vary. Secondly, the silicone coated facial profile offers something closer to a human subject compared with a rigid mannequin, however the uniform compliance does not fully represent variations in soft-tissue on a person’s face. Finally, refinement of post-processing techniques is required to better qualify the severity and intensity of vapour release allowing for greater analytical detail. Nevertheless, these results show fully-customised and semi-customised designs produce a good facial seal when applied to an individual thus supporting both approaches in providing well-fitting and effective devices.

### Prototype fit testing

**Figure 7:**
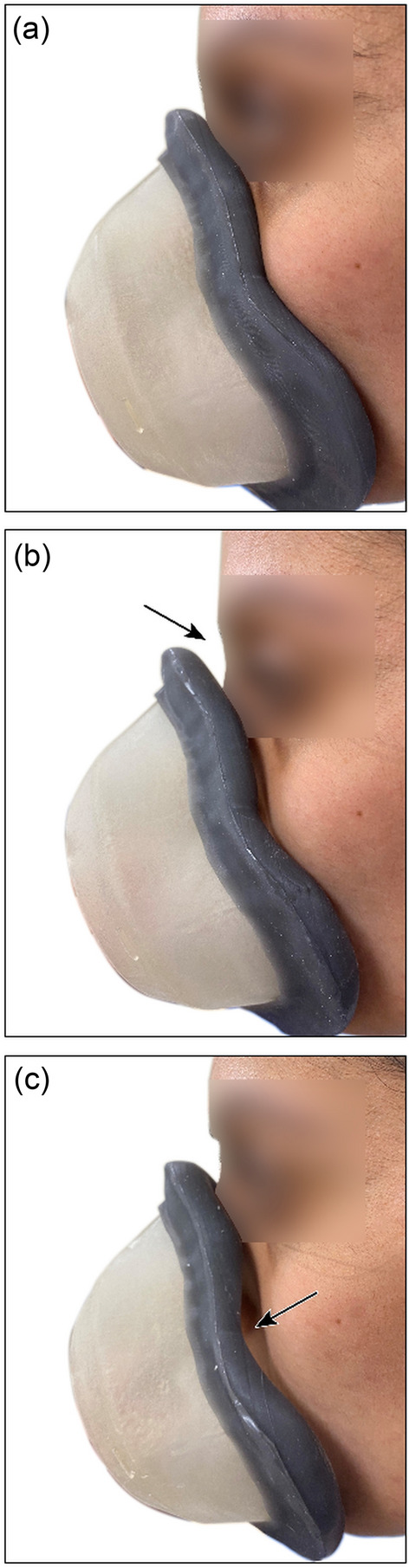
Figure showing (**a**) best matching eye-to-chin and nasal profiles (**b**) a nasal profile mismatch leading to lifting of the device away from the nose and loss of seal and (**c**) a combination of eye-to-chin and nasal profile mismatches leading to obvious gapping from the cheeks. Informed consent by the participant was obtained to publish these images.

All volunteers passed the qualitative fit test using prototype customised devices providing further confidence in the design and customisation methodology. Figure 7a shows photographs of a volunteer wearing a correctly fitted device whilst Fig. 7b shows a device with correct eye-to-chin dimension but incorrect nasal profile and Fig. 7c shows a device with an incorrect combination of eye-to-chin dimension and nasal profile leading to a significant mismatch. Regions of poor fit are indicated by arrows.

### The route to commercial product

Although the viability of the customisation methods from 3D scan data have been proven using a prototype design, development of a commercial product that exploits these methods requires broader considerations, refinement, and potentially further development of the techniques. Extraction of facial parameters is currently limited to facial size and nasal profile, however any device development would require examination of a larger sample dataset. This would further guide the design around the non-customised features, for example the cheeks where soft tissue provides more compliance, and identify other key regions where these techniques could be applied. For example, a larger dataset may reveal that parametric extraction around the zygomatic arch or facial width is necessary to fit a greater proportion of users.

Critically however in exploring these novel methods, the balance between a bespoke product and the practicalities of mass manufacture have been considered at all stages. Any marketable device would likely need to be injection moulded to be viable from a production rate and unit cost point of view. The limited number of design variations for the semi-customised and hard shell of the fully customised approach are currently limited to three and nine for the shell and seal respectively with a single common front cap. Whilst this would result in higher tooling costs compared to a single design, it is still not seen as a prohibitive number of variations given the potential benefits to the user. Furthermore, the simple Gaussian curve used to tailor the nasal profile could be achieved using inserts in to common seal tooling allowing for cost savings in tooling or expansion of the range of profiles. The rapid and automated evaluation of the scan data quickly identifies, and limits, the labour intensive and expensive fully-customised approach to those users where it is necessary.

Finally the appropriate regulatory standard for half-face respirators, British Standard EN 149^28^, presents a unique barrier to market for customised respiratory protection. In its current form the standard does not address the certification of a customised device. Any successful commercialisation of these methods in to an FFP3 rated device on the market would undoubtedly require as a minimum clarification from the regulatory body and potentially amendments to the standard.

## Conclusions

A viable workflow for both semi-customised and fully customised respirator devices has been presented driven by readily available 3D scanning technologies. This approach aims to address the current shortcomings in traditional mask fit failure rates and user comfort through customisation and material selection.

Extraction of design relevant facial parameters from 3D scan data has been shown to provide a route of rapid evaluation and categorisation of user faces. This semi-customised approach balances the need for customisation with that of large scale production making such devices practically viable. From this study, nine design variants have been identified encompassing 87.5% of participants based on facial size. This process further enable viability of fully customised devices by targeting only users falling outside standard sizes where it will provide the most benefit.

Physical testing using a novel flow visualisation method has validated the approach and illustrated the difference between correctly and incorrectly customised masks for both design routes. This is further supported by standard qualitative fit testing of semi-customised prototypes on a subset of five volunteers.

Compliance of the silicone interface material not only ensures a comfortable facial interface for the user, but also provides the necessary properties to mitigate the mismatch between the true and approximated nasal profile proving that effectiveness is not only a matter of design, but also material selection.

Customisation of respiratory protection has not only been proven feasible with existing technology but further development is essential to improve the fit, function, and consequently the protection that can be offered to frontline workers during the current pandemic.

## Data Availability

3D facial images will not be made available. However, extracted anthropometric data and other datasets generated during and/or analysed during the current study are available from the corresponding author on reasonable request.
